# Volatile Molecules Secreted by the Wheat Pathogen *Parastagonospora nodorum* Are Involved in Development and Phytotoxicity

**DOI:** 10.3389/fmicb.2020.00466

**Published:** 2020-03-25

**Authors:** Mariano Jordi Muria-Gonzalez, Yeannie Yeng, Susan Breen, Oliver Mead, Chen Wang, Yi-Heng Chooi, Russell A. Barrow, Peter S. Solomon

**Affiliations:** ^1^Research School of Biology, ACT, Australian National University, Canberra, ACT, Australia; ^2^Department of Oral Biology and Biomedical Sciences, MAHSA University, Selangor, Malaysia; ^3^School of Molecular Sciences, University of Western Australia, Perth, WA, Australia; ^4^Graham Centre for Agricultural Innovation, Charles Sturt University, Wagga Wagga, NSW, Australia; ^5^Plus 3 Australia Pty Ltd., Hawker, ACT, Australia

**Keywords:** wheat pathogens, volatiles, disease, sesquiterpenes, *Parastagonosopora nodorum*

## Abstract

Septoria nodorum blotch is a major disease of wheat caused by the fungus *Parastagonospora nodorum*. Recent studies have demonstrated that secondary metabolites, including polyketides and non-ribosomal peptides, produced by the pathogen play important roles in disease and development. However, there is currently no knowledge on the composition or biological activity of the volatile organic compounds (VOCs) secreted by *P. nodorum*. To address this, we undertook a series of growth and phytotoxicity assays and demonstrated that *P. nodorum* VOCs inhibited bacterial growth, were phytotoxic and suppressed self-growth. Mass spectrometry analysis revealed that 3-methyl-1-butanol, 2-methyl-1-butanol, 2-methyl-1-propanol, and 2-phenylethanol were dominant in the VOC mixture and phenotypic assays using these short chain alcohols confirmed that they were phytotoxic. Further analysis of the VOCs also identified the presence of multiple sesquiterpenes of which four were identified via mass spectrometry and nuclear magnetic resonance as β-elemene, α-cyperone, eudesma-4,11-diene and acora-4,9-diene. Subsequent reverse genetics studies were able to link these molecules to corresponding sesquiterpene synthases in the *P. nodorum* genome. However, despite extensive testing, these molecules were not involved in either of the growth inhibition or phytotoxicity phenotypes previously observed. Plant assays using mutants of the pathogen lacking the synthetic genes revealed that the identified sesquiterpenes were not required for disease formation on wheat leaves. Collectively, these data have significantly extended our knowledge of the VOCs in fungi and provided the basis for further dissecting the roles of sesquiterpenes in plant disease.

## Introduction

The Dothideomycete fungus *Parastagonospora nodorum* is the causal agent of Septoria nodorum blotch, a significant foliar global disease of wheat. Once considered a simplistic pathogen that caused disease through the secretion of lytic enzymes, seminal studies over the last decade have demonstrated that *P. nodorum* facilitates disease through the use of small proteins called effectors (Oliver et al., 2012). To date, three effectors from *P. nodorum* have been described, ToxA, Tox1 and Tox3 (Friesen et al., 2006; Liu et al., 2009, Liu et al., 2012). Each of these proteins interact in a gene-for-gene for manner with specific cognate susceptibility genes in the host leading to host cell death and disease. More recent studies have demonstrated that as well as inducing necrosis, each of these effectors appears to also function in repressing host defence responses highlighting the complex nature of this interaction (Breen et al., 2016; Liu et al., 2016, McDonald and Solomon, 2018).

However, it has been recently shown that ToxA, Tox1, and Tox3 are not the only molecules responsible for *P. nodorum* to successfully infect wheat (Tan et al., 2014, 2015). Tan et al. (2015) used a reverse genetic approach to generate a strain of *P. nodorum* lacking each of the effector genes and showed that the resulting mutant, albeit being less pathogenic, retained the ability to cause disease (Tan et al., 2015). Indeed, recent studies have examined the role of several polyketide secondary metabolites synthesized by *P. nodorum* and shown that some have a role in facilitating disease on wheat (Chooi et al., 2015a, b, 2017, Li et al., 2018). However, there are many more secondary metabolites encoded for within the *P. nodorum* genome that potentially play a role in the interaction of the pathogen with its host (Chooi et al., 2014; Muria-Gonzalez et al., 2015).

Another group of molecules that have yet to be characterized in terms of their role or requirement in septoria nodorum blotch are the volatile organic compounds (VOCs). VOCs are small carbon-based molecules that readily evaporate and are ubiquitously produced by most forms of life (Kesselmeier and Staudt, 1999). It has been proposed that VOCs play important roles as signals in inter and intra-organismic interactions which surpasses the involvement of other diffusible molecules (Kanchiswamy et al., 2015). Microorganisms are known to be a rich source of VOCs displaying antibacterial, antifungal, and phytotoxic properties, but also acting as chemical cues that help structuring microbial communities (Kanchiswamy et al., 2015). VOCs typically produced by microorganisms are complex blends of chemicals. The composition and role of VOCs though in fungi, particularly plant pathogens, are poorly understood.

To address this knowledge gap, we firstly explored the biological activity of the VOCs emitted by *P. nodorum* and undertook an initial identification and characterization of the major components. As a result of this, several sesquiterpene molecules were identified and the genes required for their synthesis characterized. This study has shed further light on the chemical diversity synthesized by these fungi and has raised further questions as to the roles of small molecules generated by this devastating wheat pathogen.

## Materials and Methods

### Volatile Compound-Mediated Growth Competition Assays

To test potential activities of *P. nodorum* VOCs, four fungi were used for growth antagonist assays: *P. nodorum, Fusarium oxysporum* f. sp. *lycopersici, Eutiarosporella tritici-australis* and *Zymoseptoria tritici*. *Escherichia coli, Pseudomonas syringae, Sinorhizobium meliloti*, and three other bacteria isolated from within surface sterilized wheat seeds, *Bacillus cereus*, *Sphingobacterium multivorum*, and *Flavobacterium* sp. were also used for the growth assays.

In one side of a segmented Petri dish (9 cm diameter), 25 μl of a *P. nodorum* spore solution (1 × 10^6^ spores/ml) was inoculated on Fries agar (1.5%) (Supplementary Table S1) and incubated at 22°C in 12-12 h dark and light. After two weeks, the other compartment of the segmented Petri dish was inoculated with the test organisms. The fungi were inoculated onto potato dextrose agar (PDA), the bacteria on Lysogeny broth (LB) agar. Water agar (1%) was used for the germination of wheat (*Triticum aestivum* cv. Grandin) and *Medicago truncatula* for which 8 and 10 seeds per plate were used respectively (Supplementary Table S1). Plates were sealed with parafilm after the test organisms were inoculated. The effect of the VOCs was then visually monitored daily.

To assess the phytotoxic and fungistatic effect of 2-methyl-1-propanol, 2-methyl-1-butanol, 3-methyl-1-butanol and 2-phenylethanol, each of the compounds was placed on a 1 cm^2^ filter paper on a section of a segmented Petri dish. On the other half of the dish was placed either wheat seeds or *P. nodorum* was inoculated, on the appropriate medium as described above. An additional treatment was also performed containing a mix of these compounds in the proportions found in the chromatographic analysis of *P. nodorum* VOCs. 1 mM of each of the pure compounds (74 to 122 ppm) and 100 ppm for the mix were used in these assays, considering a free internal volume of the petri dish of 48.9 cm^3^ (the total volume minus 15 ml of test medium). Plates were sealed using parafilm immediately after the compound was added, however it cannot be excluded that the molecules did not undergo some degree of diffusion through the membrane.

Each experiment was repeated twice using at least three Petri dishes per treatment. The number of seeds germinated per plate as well as radicle and coleoptile lengths were recorded and the average and standard deviation were calculated. *T*-tests were performed comparing each treatment against the control to determine statistical significance. Qualitative data was not statistically evaluated.

### Volatile Molecule Analysis

To identify the individual components of the VOCs, slanted Fries agar head-space (HS) vials (20 ml) were inoculated with *P. nodorum*. Vials with cotton stoppers were incubated for one week at 22°C in 12-12 h dark and light cycles. Vials were sealed with silicon/Teflon septa crimp caps 24 h prior to the analysis. Three mock-inoculated vials were used as controls. To calculate the retention indices, 5 μl of an alkane mix at 20 ppm in CH_2_Cl_2_ was added to a HS vial (Kováts, 1958; van Den Dool and Dec Kratz, 1963). To confirm the identity of ethyl acetate, 2-methyl-1-propanol, 2-methyl-1-butanol, 3-methyl-1-butanol and 2-phenylethanol within the fungal VOCs, a mix following the proportions found in solid phase micro-extractions in line with a gas chromatography-mass spectrometry (SPME-GC-MS) analysis of the fungal cultures (10:14:26:53:11 respectively) was prepared using pure compounds and 1 μl of the mix was added to a HS vial.

To evaluate the *in planta* production of sesquiterpenes, the distal 5 cm of the second leaf of 2-week old wheat seedlings were excised and sprayed with a 1 × 10^6^ *P. nodorum* spores/ml solution containing 0.02% tween 20. The cut end of each leaf was embedded in a HS vial containing 2 ml of water agar (1%). Vials were closed with silicon/Teflon septa crimp caps and incubated for 3 days at 22°C in 12-12 h dark and light cycles. Three mock-inoculated samples were used as controls.

The SPME-GC-MS analyses were performed in an Agilent 7890A gas chromatograph coupled to a single quadrupole Agilent 5975 mass spectrometer using a Gerstel MPS 2XL autosampler. The column for the analyses was a Varian CP9013-1Factor 4 5 m s 350°C: 40 m x 250 (μm x 0.25 (μm. Elution was performed with He flow at 1.5 ml/min and temperature programed from 40°C (hold 3 min) to 180°C at 4°C/min and then to 220°C (hold 5 min) at 10°C/min. The mass spectrometer was operated in the electron ionization (EI) mode with ionization energy of 70 eV and scanning the mass range of *m/z* 40-600. Temperatures were set to: GC inlet, 240°C; GC transfer line, 240°C; MS source, 200°C; and quadrupole 250°C. Volatiles were adsorbed onto a SPME fiber coated with divinylbenzene/carboxen/polydimethylsiloxane (DVB/CAR/PDMS) (1 cm, 23 Ga, 50/30 μm film thickness, Supelco) for 120 min at 30°C after a 5 min equilibration. The fiber was desorbed in the injector at 240°C (splitless mode 2 min). The fibers were conditioned by keeping them in the GC injector at 240°C for 10 min.

All GC-MS experiments were done in triplicate. Data was acquired using MSD ChemStation E.02.01.1177 (© Agilent Technologies, Inc.). Analysis of the data was performed using ChemStation and MS Search NIST Mass Spectral Search Program [Version 2.0g] for the NIST/EPA/NIH Mass Spectral Library [NIST Standard Reference Database 1A Version NIST 11] build May 19 2011 (© National Institute of Standards and Technology).

### Disruption of *P. nodorum* Sesquiterpene Synthases

*Parastagonospora nodorum* sesquiterpene synthases (Sts), SNOG_03562 (Sts1), and SNOG_04807 (Sts2), were individually disrupted in *P. nodorum* wild type (SN15) by split marker homologous recombination of a phleomycin resistance cassette. 1.5 Kb 5’ and 3’ flanking regions for each gene were amplified from *P. nodorum* genomic DNA (primers in Supplementary Table S2). The phleomycin resistance gene was amplified as two overlapping amplicons, Phl and Leo, from the pAN8-1 plasmid (primers in Supplementary Table S2) (Mattern et al., 1988). 5’ flanks were PCR fused to *Leo* while 3’ flanks were PCR fused to *Phl* (primers in Supplementary Table S2). *P. nodorum* was then transformed by a PEG-protoplast method as previously reported (Solomon et al., 2004).

To assess the copy number of the phleomycin cassette in the transformants, qRT-PCR primers were designed for the phleomycin resistance gene; elongation factor 1α, actin and SnToxA primers were used to normalize the data (primers in Supplementary Table S2). As a phleomycin single copy reference, a *tox3* knock out strain was used (Tan et al., 2014).

### Characterization of *P. nodorum* Sesquiterpenes

To isolate and characterize the product of Sts1 and Sts2, the coding sequences were cloned into the linearized plasmid backbone (XW55) from YEplac-ADH2p (primers in Supplementary Table S2) (Lee and DaSilva, 2005, Chooi et al., 2015a). *In vivo* yeast recombination cloning using each gene and XW55 was performed with the Frozen-EZ Yeast Transformation II^TM^ Kit (Zymo Research, Irvine, CA) and competent *Saccharomyces cerevisiae* BJ5464-NpgA according to manufacturer’s protocol. Positive transformants were selected by PCR from colonies grown on synthetic dropout agar lacking uracil.

Medium scale *P. nodorum* fermentations for the isolation of α-cyperone were performed using 8 l of liquid Fries medium inoculated with 4 × 10^6^ spores. Cultures were incubated in the dark during 10 days at 22 °C and shaking at 120 rpm.

For the isolation of acora-4,9-diene, and eudesma-4,1-diene and β-elemene, transformed *S. cerevisiae* harboring *Sts1* or *Sts2* were inoculated in in 6 ml synthetic dropout agar lacking uracil (Supplementary Table S1) for 72 h at 28°C 200 rpm. Each of these seed cultures was used to inoculate 5 l YPD broth (Supplementary Table S1) and incubated 90 h at 22°C at 200 rpm.

Fungal cultures were lyophilized and low to medium polarity compounds were extracted with dichloromethane. Yeast cultures were centrifuged and the cells subjected to acetone extraction. Both, dichloromethane and acetone were evaporated in a rotary evaporator. The sesquiterpenes from the extracts were isolated by acetonitrile/hexane partition. Sesquiterpenes were purified by SiO_2_ hexane flash chromatography followed by C18 water/acetonitrile flash chromatography. Purity of terpenes was assessed by GC-MS. Isopentane was used to recover the terpenes from the water-acetonitrile mixture.

GC-MS^2^ was performed to identify β-elemene and α-cyperone by comparison with standards in a Finnigan TraceGC ultra (Thermo Scientific) coupled to an iontrap Finnigan Polaris Q (Thermo Scientific) mass spectrometer. β-elemene was injected onto a BPX70 30 m × 0.25 mm id (SGE Analytical Science) while α-cyperone in a Varian CP9013-1Factor4 5 ms column, which were eluted with He (inlet pressure 15 psi; injection port 200°C; interface 250°C; source 200°C). For β-elemene the column was temperature programed from 60°C (hold 1 min) to 100°C at 25oC/min, then to 150 at 10 oC/min, and finally to 240°C at 10°C/min (hold 3.5 min); for α-cyperone the program started at 60°C (hold 1 min) to 200°C at 30°C/min, then to 220 at 3°C/min, and finally to 325°C at 30°C/min (hold 1 min). The mass spectrometer was operated in the electronic ionization (EI) mode with ionization energy of 70 eV, scanning the mass range of *m/z* 50–450. For MS/MS experiments, the precursor ions were selected with a peak width of 1.0 amu over 12ms. The ions were excited at 1 V for 15 ms with *q* = 0.3 and the products scanned over a mass range of *m/z* 100–250. Data analysis was performed employing Xcalibur^TM^ 1.4 (©Thermo Scientific) software.

The eudesma-4,11-diene (1) and the acora-4,9-diene (4) were dissolved in CDCl_3_ and analyzed by NMR. ^1^H NMR, ^13^C NMR, HSQC, and HMBC were performed in an Avance^TM^III HD 300 MHz NanoBay NMR device (Bruker).

### *P. nodorum* Infection Assays

To test the requirement of *Sts* genes for infection, the mutants of *P. nodorum* lacking the *Sts1* and *Sts2* genes were inoculated onto the second leaf of two-week-old wheat seedlings (cv. Axe) which was attached to a styrofoam platform using double sided sticky tape and spayed with a spore solution (1 × 10^6^ spores/ml containing 0.02% tween 20). 0.02% tween 20 was used as control. Inoculated seedlings were incubated for 48 h at 22°C in a dark moisture chamber. After the initial 48 h incubation, the inoculated plants were grown at 85% humidity, 20°C during the day and 12°C at night with 16-8 h light/dark cycles. The leaves were collected at five days post inoculation to evaluate the disease. These assays were performed twice with a minimum of four leaves per treatment used.

## Results

### *Parastagonospora nodorum* VOCs Have Phytotoxic, Antibiotic, and Self-Inhibitory Properties

**FIGURE 1:**
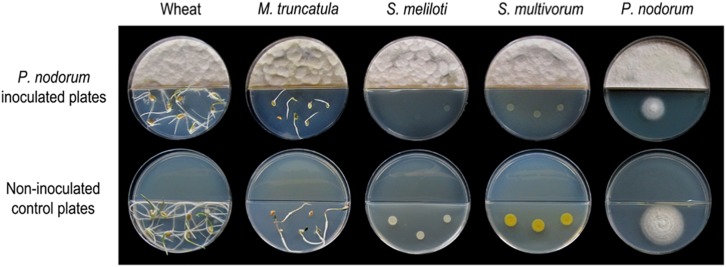
Split plate assays of the effect of VOCs produced by wild type *P. nodorum* (SN15). The top row of plates show the inhibition caused by *P. nodorum* VOCs on wheat and *Medicago* seeds, *S. meliloti, S. multivorum*, and *P. nodorum*. The bottom row of plates shows the growth of each of the test organisms in the absence of *P. nodorum*.

To assess if volatile emissions of *P. nodorum* harbor bioactive VOCs, split plate assays were performed to evaluate a series of biological activities including phytotoxicity, fungitoxicity and bactericidal (Figure 1). Segmented Petri dishes were used to prevent molecules diffusing through the media and ensure that any observable activities could be solely attributed to the volatile compounds. To assay for phytotoxicity, seeds from the host of *P. nodorum*, wheat *cv.* Grandin, and a dicotyledonous plant, *Medicago truncatula*, were used. *P. nodorum* VOCs had a strong effect on the radicle elongation and hypocotyl or coleoptile growth which were significantly reduced when the seeds were germinated in the presence of *P. nodorum* but appeared unaffected in the control plates (no fungal inoculation). The effect on bacteria was mixed as there was no observable impact on the growth of a variety of different strains including *Escherichia coli*, *Pseudomonas syringae*, *Bacillus cereus*, or *Flavobacterium* sp. in the presence of the fungal VOCs (Supplementary Figure S1). In contrast, there was a strong reduction in the growth of the nitrogen-fixing bacterium *Sinorhizobium meliloti* and also *Sphingobacterium multivorum* when *P. nodorum* was cultured in the same Petri dish (Figure 1). There was no apparent impact on the growth of any of the fungi tested when grown with *P. nodorum* with the exception of the apparent self-inhibition of *P. nodorum* growth (by its own VOCs) (Figure 1 and Supplementary Figure S1).

### The Major *P. nodorum* VOCs Are Short Chain Alcohols

To dissect the chemical basis of the bioactivities described above, the identities of the volatile molecules were determined using a combination of solid phase micro-extractions (SPME) from the headspace (HS) of ten days old fungal cultures in slanted Fries agar vials and subsequent analysis by gas chromatography-mass spectrometry (GC-MS) and spectral comparison against pure standard and the NIST library. Within the *P. nodorum* VOCs mixture, several alcohols and esters were identified as being the most prominent signals (percentage of area of the whole chromatogram) (Table 1): 3-methyl-1-butanol (representing 5.36% of the VOCs mixture), 2-methyl-1-butanol (2.6%), 2-methyl-1-propanol (1.43%) and 2-phenylethanol (1.13%). Many other volatile molecules were also identified in peaks with smaller areas. The polyketide mellein (0.9%) was also detected along with some sesquiterpenes of which two were putatively identified as β-elemene and eudesma-4,11-diene.

**TABLE 1:** VOCs identified from *P. nodorum* grown on Fries media using HS-SPME-GC-MS.

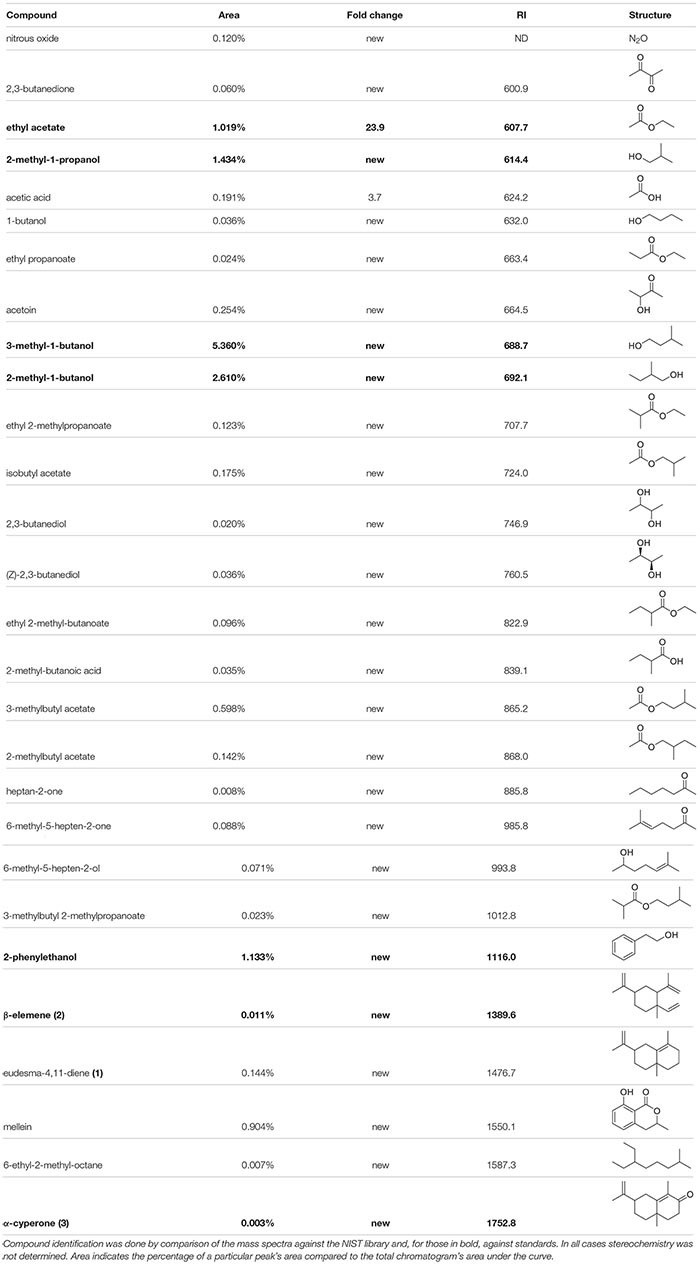

### The Four Most Abundant *P. nodorum* VOCs Are Phytotoxic

**FIGURE 2:**
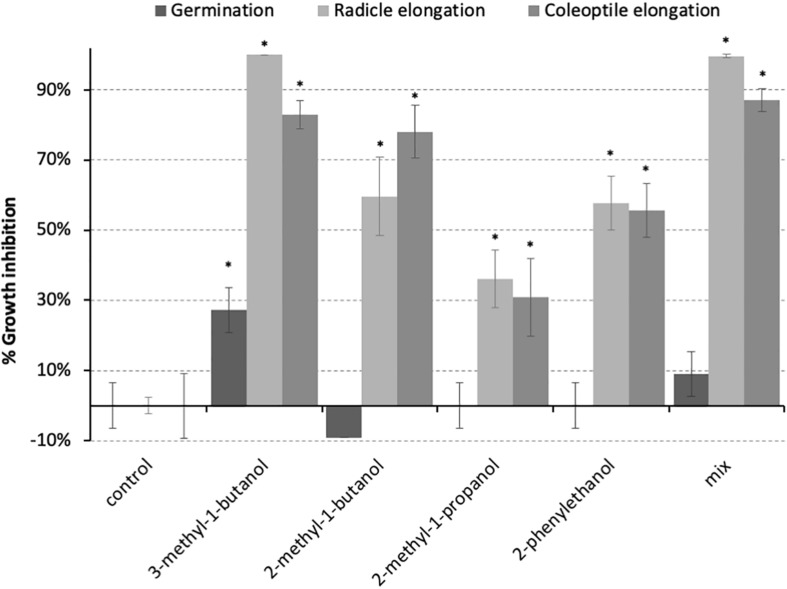
Inhibition of wheat seedling development by the predominant VOCs produced by *P. nodorum*. The bars represent the inhibitory activity on the developmental stage. 3-methyl-1-butanol, 2-methyl-1-butanol, 2-methyl-1-propanol, and 2-phenylethanol were tested at 1 mM. Error bars show the standard deviation and asterisks indicate statistically significant differences compared to the control (*p* < 0.05).

The activity of the four most prominent volatile molecules identified in the chromatograms from the head space of *P. nodorum* (3-methyl-1-butanol, 2-methyl-1-butanol, 2-methyl-1-propanol, 2-phenylethanol) were assayed to assess their impact on the growth of *P. nodorum* and wheat seedling development. These compounds were tested independently at an atmospheric concentration of 1 mM. Additionally, a mixture of these compounds following the *in vitro* proportions, was prepared and tested at 100 ppm. Neither the independent pure VOCs nor the mixture had any effect on *P. nodorum* growth suggesting that these molecules are not responsible for the inhibitory effect described above (data not shown). In contrast, a 27% decrease in germination was observed when wheat seeds were exposed to 3-methyl-1-butanol, although no other treatment had a significant effect on germination (Figure 2). However, both radicle and coleoptile elongation were repressed in all treatments; 3-methyl-1-butanol showed the greatest inhibition (100% and 83% respectively) while 2-methyl-1-propanol showed the least inhibition (36% and 31% respectively).

### *In planta* Production of Sesquiterpenes

**FIGURE 3:**
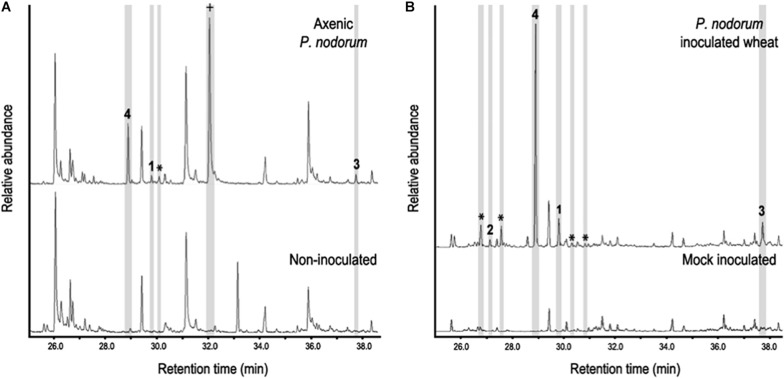
**(A)** Sesquiterpene profile of *P. nodorum* growing on Fries media (upper panel) and uninoculated Fries media (lower panel). **(B)** The sesquiterpene profile of wheat leaves infected with *P. nodorum* (upper panel) and mock inoculated wheat leaves (low panel). Compounds 1-4 are shown and other sesquiterpenes peaks are stared (*). Mellein is indicated as a cross (+).

In addition to the bioactive short chain alcohols, we were also interested in the presence of the sesquiterpenes found in the axenic culture VOCs. To determine if these molecules played a potential role in disease development, the production of sesquiterpenes was assayed for during infection and compared to those produced in axenic culture. VOCs were extracted from vials containing either infected leaves or the fungus grown axenically and analyzed by HS-SPME-GC-MS. Interestingly the same sesquiterpenes produced *in vitro* by *P. nodorum* were also found *in planta* as well as others not previously observed (Figure 3). Eudesma-4,11-diene (sesquiterpene 1), β-elemene (sesquiterpene 2) and α-cyperone (sesquiterpene 3) were putatively identified by comparing the acquired data against the NIST database. Another interesting compound was sesquiterpene 4, the most abundant sesquiterpene detected from *P. nodorum*, in Fries cultures and in wheat leaves. However, despite a fragmentation pattern and molecular weight typical of a sesquiterpene, its match against entries present in the NIST library was not high enough to confidently assign a possible identity.

### The Biosynthetic Genes of *P. nodorum* Sesquiterpenes

The *P. nodorum* genome encodes three sesquiterpene synthases (Chooi et al., 2014), *Sts1*, *Sts2*, and *Sts3*. Previous studies have demonstrated that *Sts1* and *2* are expressed during infection, but not *Sts3*. Furthermore, analysis of the *Sts3* gene sequence revealed that it appears truncated implying that it isn’t functional and thus it was not considered for further study (Ipcho et al., 2012). To directly link the molecules identified above to the genes, *Sts1 and Sts2* were disrupted individually in the *P. nodorum* genome through homologous recombination. Disruption cassettes were constructed to independently replace *Sts1* and *Sts2* with a phleomycin resistance marker. *P. nodorum* was transformed and positive colonies were selected from phleomycin-containing plates. Correct disruption of the genes was verified by PCR and strains containing a single copy of the disruption cassette were selected by qPCR.

**FIGURE 4:**
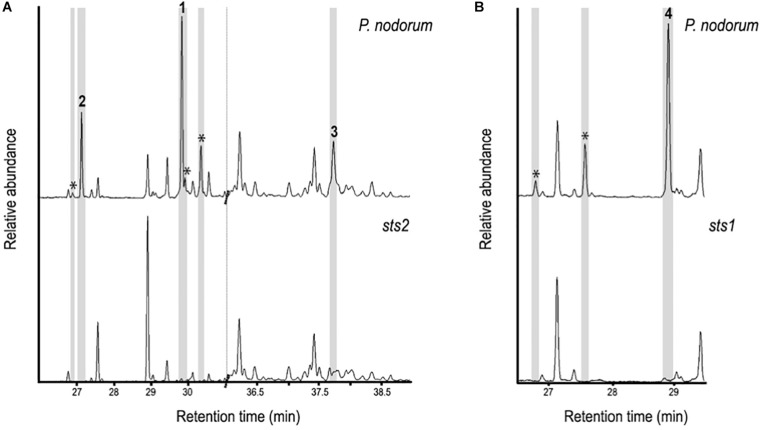
GC-MS chromatograms comparing sesquiterpenes in the extracts from wild-type *P. nodorum* to mutant strains lacking Sts2 **(A)** and Sts1 **(B)**. The presence of 1 – 4 in the wild-type strain is highlighted as well as other sesquiterpenes peaks which are highlighted (*).

Single copy transformants of *P. nodorum* strains lacking *Sts1* (*sts1*) and *Sts2* (*sts2*) were selected for further analysis by HS-SPME-GC-MS (Figure 4). The signals of sesquiterpenes 1, 2, and 3 along with three other unidentified sesquiterpenes present in wild-type *P. nodorum* were absent in the *sts2* mutants indicating this gene codes for the core biosynthetic enzyme of the three putative sesquiterpenes plus some other sesquiterpene structures (same mass and similar fragmentation pattern). The mutants lacking *sts1* were missing three unidentified compounds putatively identified as sesquiterpenes, including sesquiterpene 4, suggesting that *Sts1* is responsible for its synthesis.

### Heterologous Expression of *Sts1* and *Sts2* Reveals Its Prolificity and Allows the Sesquiterpenes Isolation

**FIGURE 5:**
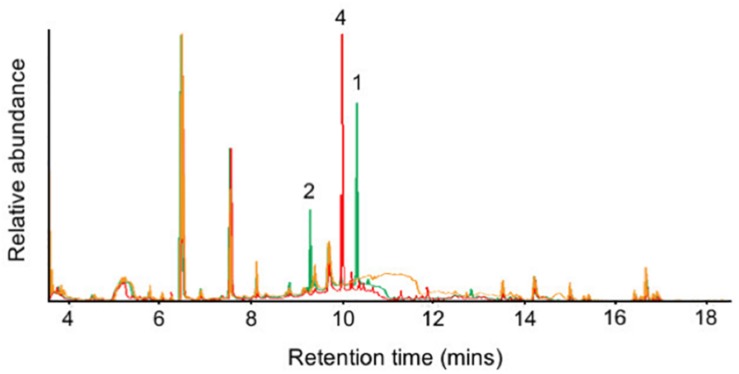
GC-MS chromatograms of the extracts from the *Sts1* and *Sts2* heterologous expression in yeast. Overlapped chromatograms of extracts from yeast harbouring *Sts1* (red), *Sts2* (green) and an empty vector (orange). The presence of 1, 2, and 4 is indicated.

To confirm the identity of these sequiterpenes, an isolation from medium scale fermentation of *P. nodorum* in Fries media was undertaken. Sesquiterpene 3 was isolated by silica flash chromatography followed by C18 flash chromatography. However, the isolation of sesquiterpenes 1, 2, and 4 was not achieved. Consequently, the *Sts1* and *Sts2* genes were heterologously expressed in yeast to confirm 1 and 2 as eudesma-4,11-diene and β–elemene respectively. Acetone extracts from small scale cultures of the yeast strains harboring the sesquiterpene synthases were analyzed by GC-MS and the production of 1, 2, and 4 by the heterologous expression of *Sts2* and *Sts1* was confirmed (Figure 5). Interestingly, sesquiterpene 3 (putatively α-cyperone) was not detected, suggesting it is modified post synthesis.

Sesquiterpenes 1 and 2, and 4 were isolated by silica followed by C18 flash chromatography of acetone extracts from medium scale YPDA fermentations of yeast carrying *Sts2* and *Sts1* respectively. Subsequent GC-MS analysis of the sesquiterpene fractions confirmed that 4 is the major product of Sts1 but also that 14 other unidentified terpenes are synthesized by the same enzyme (Supplementary Figure S2). Similarly, the predominant sesquiterpene produced by Sts2 is 1 along with 10 other molecules including compound 2 (Supplementary Figure S3).

### The Identity of the *P. nodorum* Sesquiterpenes Are Confirmed by MS^2^ and NMR

**FIGURE 6:**
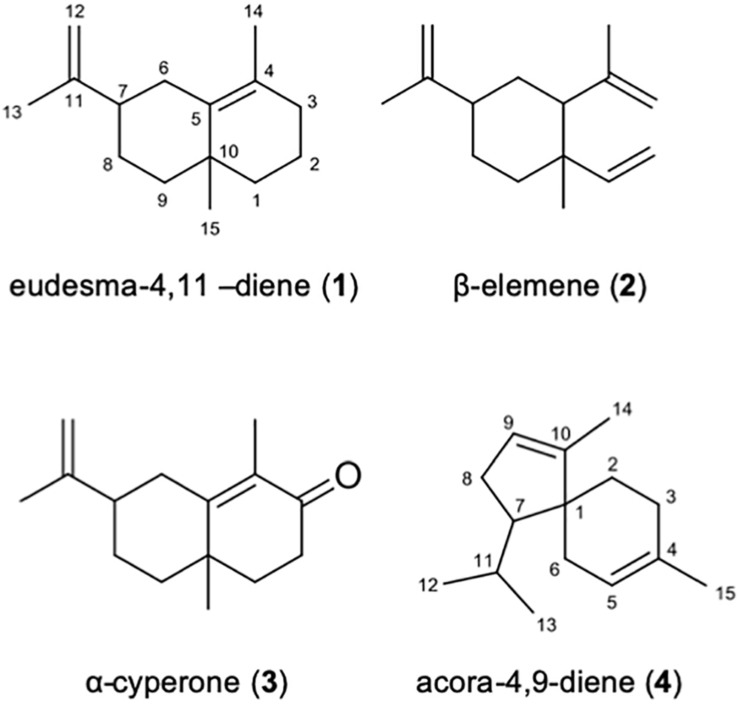
Identified sesquiterpenes present in *P. nodorum* VOCs.

Commercial standards of β-elemene and α-cyperone were purchased and analyzed by GC-MS^2^ to confirm the identities of sesquiterpenes 2 and 3. Identical retention times in addition to a comparison of the MS^2^ fragmentation profiles that demonstrated a complete overlap of the major ions present in the standards compared to the extracted samples confirmed the identities of 2 and 3 (Figure 6 and Supplementary Figures S4, S5).

The isolated sesquiterpene 1, the putative eudesma-4,1-diene, and unknown sesquiterpene 4 were subjected to ^1^H NMR, ^13^C NMR, HMBC and HSQC experiments (Supplementary Figures S6–S13). Supplementary Tables S3, S4 present the assignments and chemical shifts (δ) for all carbons and the corresponding hydrogens as well as the correlations obtained from the HMBC experiment for sesquiterpene 1 and sesquiterpene 4 respectively. The identity of sesquiterpene 1 was confirmed to be eudesma-4,11-diene, and sesquiterpene 4 is proposed to be acora-4,9-diene (Figure 6) based on comparison to previously reported NMR data (Iwabuchi et al., 1989; Chen and Lin, 1993).

### *Sts1* and *Sts2* Are Not Required for Bacterial Growth Inhibition, Phytotoxicity or for the Infection of Wheat by *P. nodorum*

A segmented plate growth assay was used to determine if the molecules derived from either *Sts1* or *Sts2* are involved in the biological activities described above. As previously observed, volatiles from wild type *P. nodorum* reduced wheat seed germination and inhibited the growth of *S. multivorum* as well as self-growth. The growth of both the *sts1* or *sts2* mutants caused the same phenotypic effects as the wild type inferring that the products of either gene are not responsible for these inhibitory activities (data not shown).

The role of the Sts1 and Sts2 terpenes in pathogenicity was evaluated by inoculating the second leaf of two-week-old wheat seedlings with wild type *P. nodorum* and each of the mutants. Symptom development for the *sts1* and *sts2* mutants was unaffected compared to the wild type suggesting that the genes do not play a role in disease as assayed in this attached leaf system (Supplementary Figure S14).

## Discussion

The roles and functions of volatile organic compounds produced by fungi are poorly understood. As such, we embarked upon a study to determine if the VOCs produced by the wheat pathogen *P. nodorum* were biologically active, and if so, resolve their identity. In this study, we have demonstrated that the wheat pathogen *P. nodorum* produces a range of VOCs that harbor intrinsic biological activities including inhibiting effects on plant seedlings, bacterial growth and also self-growth.

Initial assays clearly displayed that *P. nodorum* secretes bioactive VOCs as observed through the growth inhibition and phytotoxicity. It was also interesting to see evidence that the secreted VOCs may have a role in self growth regulation of the pathogen. Previous studies have demonstrated that volatiles can function in fungal self-inhibition of growth. For example, 1-octen-3-ol, a short chain alcohol produced by *Penicillium paneum*, and the sesquiterpene thujopsene from *Penicillium decumbes*, are known to inhibit the growth of the source fungi (Chitarra et al., 2004; Polizzi et al., 2011). Similarly, the selective inhibition of the *P. nodorum* VOC complement against bacteria observed is not without precedent. It has been previously demonstrated that compounds produced by sponge-associated Arctic microbial communities show a strong inhibitory activity against the opportunistic pathogenic bacterial *Burkholderia cepacia* complex but not to other pathogenic bacteria (Papaleo et al., 2012; Romoli et al., 2013). Why VOCs from *P. nodorum* would harbor this specificity is unclear but it has been suggested that such selectivity may be a reflection of how different organisms respond differently to the same chemical cue or alternatively it may be a consequence of possible fitness differences among individuals. Such an effect of VOCs on shaping microbial communities has been previously proposed (Tirranen and Gitelson, 2006).

An analysis of the *P. nodorum* VOC chromatograms revealed that the four most prominent compounds are well-described short chain alcohols (Table 1). 3-Methyl-1-butanol and 2-methyl-1-butanol have been previously demonstrated to inhibit the growth of the fungal pathogen *Sclerotinia sclerotiorum* (Fialho et al., 2011). Similarly, 2-phenylethanol affects gene expression and interferes in epigenetic regulation leading to the growth inhibition of multiple fungi including *Aspergillus flavus*, *Neurospora crassa* and *Penicillium* spp. (Lester, 1965; Hua et al., 2014, Liu et al., 2014). In contrast, low concentrations of 2-phenylethanol stimulates, rather than inhibits, the growth of *A. flavus*, revealing a hormetic behavior of this compound (Chang et al., 2015). These data may suggest the involvement of these short chain alcohols in metabolic regulation and could be generalized communication signals in fungi. Furthermore, considering that these molecules are produced by a broad range of organisms, one could hypothesize that communication occurs at various levels ranging from interspecific to inter-kingdom crosstalk. Interestingly, when tested in pure form or artificially blended, these VOCs had no observable effect on the development of *P. nodorum*. While there is a possibility that the tested concentration (1 mM) is not sufficient for triggering an observable effect, the most plausible explanation for the lack of response from *P. nodorum* is that other compounds we have not tested maybe involved in regulating the pathogen’s growth, either singularly, or in combination with one of the major VOCs.

The effect of the four main *P. nodorum* volatile alcohols over plant and bacterial development has also been previously described. Akin to our observations, VOCs from truffles (*Tuber* spp.) inhibited the development of *Arabidopsis thaliana* (Splivallo et al., 2007). Within the volatile emissions of tuber fruiting bodies, 3-methyl-1-butanol inhibited *A. thaliana* germination at 130 ppm while 2-phenylethanol was inhibitory at 13 ppm and caused discoloration of the cotyledons of germinated seedlings at 130 ppm (Splivallo et al., 2007).

In contrast, VOCs mixtures produced by rhizobacteria containing 3-methyl-1-butanol, 2-methyl-1-butanol and 2-methyl-1-propanol promote growth *of A. thaliana* (Ryu et al., 2003; Farag et al., 2006). It is possible that this differential effect is caused by variations in the proportion of the components of the volatilomes. It is known that differences in VOC levels in the soil correlate to changes in microbial soil populations (McNeal and Herbert, 2009). If we consider that interaction between organisms is a biological network that interweaves at different levels (Pritchard and Birch, 2011), it could be speculated that common volatile metabolites may help to coordinate the network by allowing organisms to eavesdrop on the communication signals from their neighbors (Baldwin et al., 2006; Cha et al., 2011, Caruso and Parachnowitsch, 2016). Assuming that VOCs have an active role on interspecific communication, it is important to highlight an epistemological weak point of this study; the VOCs bouquet is expected to be dynamic and depending on the challenging (test) organism, *P. nodorum* emissions may differ from the compound profile of the axenic headspace vials analyzed by GC-MS.

Together with the short chain alcohols, a suite of sesquiterpenes were also identified in the VOC mixture. The presences of eudesma-4,11-diene (1), β-elemene (2), α-cyperone (3), and acora-4,9-diene (4) were all confirmed through a combination of mass spectrometry and NMR analysis. Subsequent reverse genetics and overexpression experiments then confirmed that the sesquiterpene synthase genes in *P. nodorum*, *Sts1* and *Sts2*, were responsible for their biosynthesis. Given the presence and abundance of these molecules during infection of wheat by the pathogen, it was surprising that mutant strains of the fungus lacking these molecules appeared unaffected in terms of development or pathogenicity. Indeed, information is scarce on what precisely the functions of these sesquiterpenes identified above are.

Many plants emit eudesma-4,11-diene (1) as a minor component of biologically active VOCs mixtures or essential oils (Yoshida et al., 1967; Chu et al., 2011, Lee and Vairappan, 2011; Takahashi et al., 2011, Wang et al., 2011). While 1 is produced by some basidiomycete and ascomycete fungi and by some actinomycetes, no biological activity has been described for either fungi or bacteria (Rösecke et al., 2000; Ayoub et al., 2009, Brock and Dickschat, 2013; Rabe et al., 2013, Yuan et al., 2013). Furthermore, 1 is produced (along with other sesquiterpenes) by soldier termites from different species and *Ceroplastes ceriferu*, a scale insect (Naya et al., 1978; Braekman et al., 1993, Krasulova et al., 2012). Similarly, many plants and insects such as termites, aphids, butterflies and lady beetles, produce β-elemene (2) (Bowers et al., 1977; Baker et al., 1981, Everaerts et al., 1993; Quintana et al., 2003, Omura et al., 2006; Adio, 2009, Fassotte et al., 2014). The ascomycetes *Penicillium clavigerum*, *Penicillium roqueforti* and an endophytic *Nodulisporium* sp., and the basidiomycetes *Inonotus obliquus* and *Piptoporus betulinus*, are known to produce β-elemene (Fischer et al., 1999; Rösecke et al., 2000, Jeleń, 2002; Ayoub et al., 2009, Sanchez-Fernandez et al., 2016). However, no role for this molecule in fungi has been identified.

The distribution of α-cyperone (3) seems to be more restricted. The molecule was first identified from the rhizomes of *Cyperus rotundus*, a medicinal plant which is also classified as an invasive grass (Bradfield et al., 1936). It has been postulated that **3** is causal to the described antimicrobial, phytotoxic, insecticidal, anti-inflammatory and antimalarial activities in essential oils from *C. rotundus* (Komai et al., 1977, Dadang et al., 1996, Mojab et al., 2009; Pirzada et al., 2015). In fungi there is just one report corresponding to a stereoisomer of 3 isolated from an endophytic *Ascochyta* sp. from *Meliotus dentatus* but it has the opposite configuration to the plant isolated α-cyperone (Krohn et al., 2007). Importantly, there are no reports on what this change in stereochemistry has on the function of the molecule. *Ascochyta* and *P. nodorum* are closely related fungi so it would not be unexpected if the α-cyperone identified in this study was also the opposite stereochemistry to the plant-derived molecule. As such, it is difficult to infer what the function of α-cyperone in *P. nodorum* may be.

In contrast to the widespread occurrence of molecules 1–3 discussed above, acora-4,9-diene (4), synthesized by Sts1 in *P. nodorum* has only been found in the oils of vetiver (*Chrysopogon zizanioides*), in the seeds of carrot (*Daucus carota*), and in the glandular trichome exudates from leaves of Japanese rose (*Rosa rugosa*), with no biological activity described up to date (Kaiser and Naegeli, 1972; Hashidoko et al., 1992, Mazzoni et al., 1999).

Proposing an ecological role for Sts1 and Sts2 sesquiterpenes in the *P. nodorum*-wheat pathosystem is difficult due to the diversity of producers and reported activities of eudesma-4,11-diene (1) and β-elemene (2), the uncertainty of the *P. nodorum* α-cyperone (3) stereochemistry, the lack of information about acora-4,9-diene (4), and the absence of evident effects on pathogenicity, phytotoxicity, antimicrobial or self-regulating properties. Nonetheless, the *in planta* production of these molecules suggests its possible involvement in the fungal-plant interaction. Subtle changes in the interaction conferring some “competitive” advantage to the pathogen may not be easily detected in the laboratory pathogenicity tests which are not indicative of the full disease cycle of the pathogen.

**FIGURE 7:**
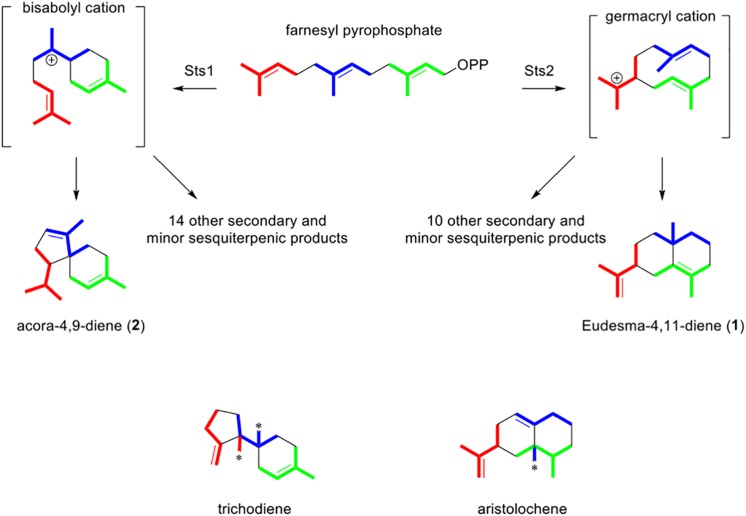
Folding pattern of farnesyl pyrophosphate (FPP) in *P. nodorum* Sts1 and Sts2. Color indicate the different isoprene units. Asterisks in methyl groups of trichothecene and aristolochene indicate a rearrangement and the color indicates to which isoprene unit it corresponds.

Aside from the ecological role of the sesquiterpenes, the linking of genes to products in this study also provides an opportunity to better understand the biosynthesis of the identified sesquiterpenes. A non-redundant BlastP analysis of Sts1 and Sts2 suggested that the two proteins are related to trichodiene and aristolochene synthases respectively (Supplementary Figure S15). These two enzymes have many similarities; in both cases the linear precursor of their products is farnesyl pyrophosphate (FPP) which loses its phosphate group and cyclizes into a cationic cyclic intermediate. The difference is the type of carbocycle produced, which depends on the tertiary structure of the enzyme. While trichodiene synthase produces a bisabolyl cation, the aristolochene synthase produces a germacranyl cation (Cane, 1990). The sequence similarity of Sts1 and trichodiene synthases is congruent with the fact that the structure of acora-4,9-diene, and the other 14 putative sesquiterpenes produced by Sts1, seems to require a bisabolene intermediate (Figure 7). Conversely, a germacranyl intermediate is the likely intermediary of eudesma-4,11-diene, β-elemene, and the other 10 products of Sts2, which corresponds to the similarity between this enzyme and aristolochene synthases (Figure 7). The generation of the multiple products by a single sesquiterpene synthase is due to the intermediaries, bisabolyl and germacranyl cations in this case, suffering spontaneous rearrangements with minimal involvement of the biosynthetic enzyme. Terpene cyclases displaying a higher control over these subsequent reactions may produce fewer structures or even a single product (Christianson, 2008). The generation of a wide spectrum of sesquiterpenes or other chemical structures increase the chances of some of these molecules having the right conformation to interact with diverse biological targets and affecting other organisms.

Even though many biological interactions are established through VOCs, the roles and synthesis of these compounds in fungi are poorly understood. In this study we have found that, in addition to the well-characterized proteinaceous effectors produced by *P. nodorum* as disease determinants, VOCs produced by this pathogen *in vitro* also trigger a response in the host plant as well as having effects on other microorganisms. Additionally, the discovery of *P. nodorum* sesquiterpenes represents the first report of terpenes in this pathogen and complementary techniques were used to link these sesquiterpene structures to their respective biosynthetic genes. Despite these advances, this study exemplifies the many unknowns that remain pertaining to VOCs in fungi and highlights their potential for future research. Unfortunately, the complexity of the volatile bouquets and the chemical signals conveyed by them represent a major challenge when teasing apart biological activities and ecological roles.

## Author’s Note

This manuscript has been released as a Pre-Print on BioRxiv (Muria-Gonzalez et al., 2019).

## Data Availability Statement

All datasets generated for this study are included in the article/Supplementary Material.

## Author Contributions

MM-G, Y-HC, and PS contributed to the design and concept of the study. MM-G, SB, OM, CW, and YY contributed to the experimentation and collection of data. Y-HC and RB contributed to data analysis. MM-G, Y-HC and PS wrote the manuscript. All authors intellectually contributed to the study and revision of the manuscript.

## Conflict of Interest

RB was employed by Plus 3 Australia Pty Ltd. The remaining authors declare that the research was conducted in the absence of any commercial or financial relationships that could be construed as a potential conflict of interest.
